# BMP9 Promotes the Proliferation and Migration of Bladder Cancer Cells through Up-Regulating lncRNA UCA1

**DOI:** 10.3390/ijms19041116

**Published:** 2018-04-08

**Authors:** Liyao Gou, Mengyao Liu, Jing Xia, Qun Wan, Yayun Jiang, Shilei Sun, Min Tang, Lan Zhou, Tongchuan He, Yan Zhang

**Affiliations:** 1 Key Laboratory of Diagnostic Medicine Designated by the Chinese Ministry of Education, Chongqing Medical University, Chongqing 400000, China; gouliyao@foxmail.com (L.G.); 2015111060@stu.cqmu.edu.cn (M.L.); xiaj101004@foxmail.com (J.X.); 17774968931@163.com (Q.W.); jiangyayundy@foxmail.com (Y.J.); ssl550140252@foxmail.com (S.S.); tm173@foxmail.com (M.T.); zhoulan0111@foxmail.com (L.Z.); 2 Molecular Oncology Laboratory, Department of Surgery, University of Chicago Medical Center, Chicago, IL 60637, USA; t_c_he@yahoo.com

**Keywords:** BMP9, *UCA1*, AKT, lncRNA, bladder cancer

## Abstract

As the most common malignant tumor of the urinary system worldwide, the bladder tumor has a high mortality rate, which is mainly due to its onset of concealment. Therefore, research into novel diagnostic markers and treatment of bladder cancer is urgently needed. BMP9 (Bone morphogenetic protein 9) is a member of BMP, which belongs to the TGF-β (transforming growth factor-β) superfamily. It has been associated with multiple tumors. We found that BMP9 is highly expressed in bladder cancer cells and it could significantly promote the proliferation and migration of bladder cancer cells. In the study of the mechanism of this effect, we found that BMP9 can increase the expression of lncRNA *UCA1* (*Urothelial cancer associated 1*) through phosphorylated AKT. The promoting effect of BMP9 on bladder cancer cells was rescued after interfering with *UCA1* in BMP9 overexpressed bladder cancer cells both in vitro and in vivo. Our research confirms that BMP9 promotes the proliferation and migration of bladder cancer cells through up-regulated lncRNA *UCA1*. It also shows that BMP9 is a novel diagnostic marker and a potential therapeutic target in bladder cancer.

## 1. Introduction

Bladder cancer has become the most common malignant tumor in the genitourinary system and the fourth leading cause of cancer-related deaths in males worldwide [1,2]. It has been reported that there are about 79,030 new bladder cancer patients and 16,870 deaths expected for 2017 in the US [3]. However, the lack of understanding of the molecular mechanism of bladder cancer development leads to the deficiency of effective treatment for bladder cancer, especially muscle-invasive bladder cancer. Approximately one quarter of newly diagnosed bladder cancer cases are muscle-invasion bladder cancer (MIBC) [4,5]. The reported five-year overall survival (OS) rate after radical or systemic platinum-based combination chemotherapy (CHT) is less than 50% [6,7]. Therefore, new molecular markers are urgently needed for the diagnosis and treatment of bladder cancer [8,9].

After the comparative sequence analysis of 256 cases of bladder mucosa specimens of database, we found that the expression of bone morphogenetic protein 9 (BMP9) was higher in both superficial and infiltrating bladder cancer cells than normal bladder mucosa, which suggests that BMP9 may be involved in the development of bladder cancer. BMP9 (also called growth differentiation factor 2) is a member of BMPs, which belongs to the transforming growth factor-β (TGF-β) superfamily [10,11]. It has a variety of biological regulatory functions including bone formation, embryonic development, tumorigenesis, and more [12,13,14]. BMP9 was reported to be a tumor suppressor in breast cancer [15,16] and lung cancer [17], but it can promote the progress of ovarian cancer [18] and liver cancer cells [19] and its function in bladder cancer has not been reported.

In this study, we found that BMP9 could promote the proliferation and migration of bladder cancer cells. This effect is consistent with its family member BMP2 and BMP7 [20]. Since a number of studies have reported that lncRNA （long non-coding RNA） is implicated in the development of bladder cancer [21,22,23], we intended to explore whether BMP9 could promote the proliferation and migration of cancer cells through lncRNAs. In the present study, we found that lncRNA *UCA1* may play a critical role in the effect of BMP9 on bladder cancer cells. *Urothelial cancer is associated 1* (*UCA1*), which is a long non-coding RNA length 1442 bp that was first cloned and identified in bladder cancer BLZ-211 cells. *UCA1* is reported to be overexpressed in bladder cancer and it promotes bladder cancer cell proliferation, migration, and invasion [24,25]. Our study confirmed that BMP9 could up regulate the expression of *UCA1* in an AKT-dependent pathway in bladder cancer cells. This may be the main reason for BMP9 promoting the proliferation and migration of bladder cancer cells. Cumulatively, these findings first demonstrate that BMP9 is a key regulator in the progress of bladder cancer cells, which highlights that it has the potential to be used as a therapeutic target in bladder cancer patients.

## 2. Results

### 2.1. The Validation of Recombinant Adenovirus BMP9 and siBMP9 

**Figure 1:**
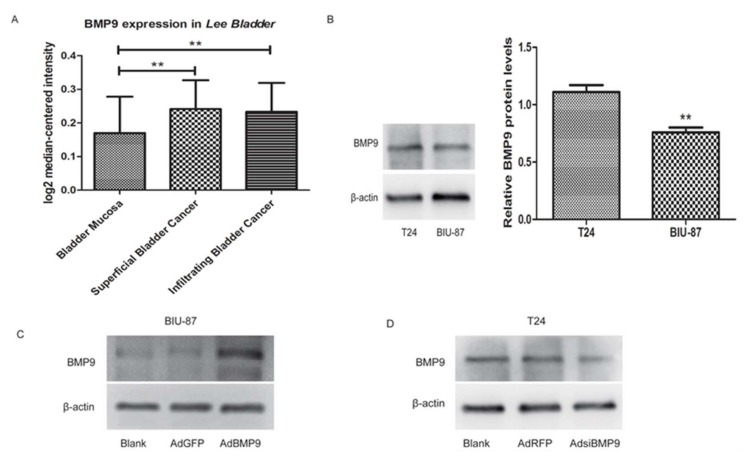
The validation of recombinant adenovirus BMP9 and siBMP9. (**A**) The expression of BMP9 in normal bladder mucosa (*n* = 126), superficial bladder cancer (*n* = 68), and infiltrating bladder cancer (*n* = 62) in the *Lee Bladder* database. *p* = 0.007; (**B**) The expression levels of BMP9 in T24 and BIU-87 cells were detected by western blot; (**C**) The BMP9 was up-regulated in BIU-87 cells after being transfected with AdBMP9 compared to the control group; (**D**) The BMP9 was down-regulated in T24 cells after being transfected with AdsiBMP9 compared to the control group. Data are shown as mean ± SD. ** *p* < 0.01.

To detect the difference in expression of BMP9 in bladder cancer and normal bladder tissues, 256 cases of bladder mucosa specimens (68 cases of superficial bladder cancer (T, Ta, Tis), 62 cases of infiltrating bladder cancer (T1–T4) and 126 cases of normal bladder) in the *Lee Bladder* database on Oncomine (available online: www.oncomine.org) were used for sequence alignment analysis. As shown in Figure 1A, the expression of BMP9 in both superficial bladder cancer and infiltrating bladder cancer are higher than in the normal, healthy bladder, which suggests that the BMP9 may participate in the development and progression of bladder cancer. The bladder cancer BIU-87 and T24 cells were used for the next experiment. First, we tested the basal expression of BMP9 on these two kinds of cells and found that its level in T24 cells was slightly higher than in BIU-87 cells (see Figure 1B). Therefore, the AdBMP9 (Recombinant adenoviruses BMP9) and AdsiBMP9 (Recombinant adenoviruses small interfering BMP9) were successfully transfected into BIU-87 and T24 cells, respectively. Figure 1C,D showed the expression of BMP9 in BIU-87 and T24 cells after transfection.

### 2.2. BMP9 Promotes the Proliferation and Migration of Bladder Cancer Cells 

**Figure 2:**
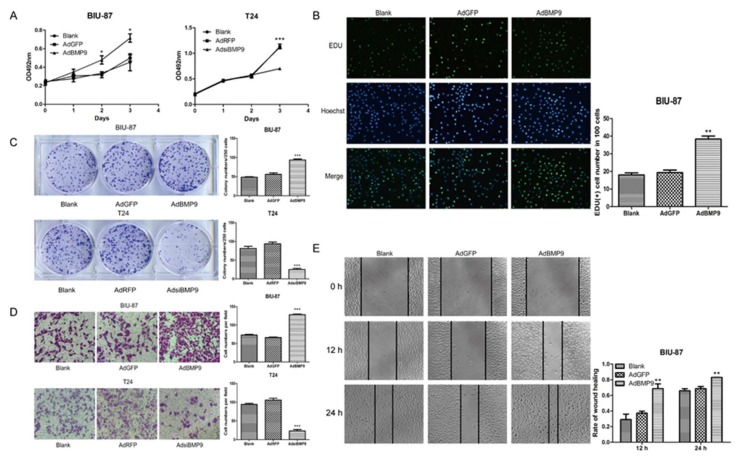
Effects of up-regulation and down-regulation of BMP9 in bladder cancer cells on cell proliferation and migration. (**A**) The cell proliferation was detected by MTT assay; (**B**) The cell proliferation was detected by EDU test. Green for proliferation cells nucleus. Blue for all living cells nucleus (400×); (**C**) The cell proliferation was detected by colony forming test. Stained with 0.1% crystal violet; (**D**) The cell migration was determined with the Transwell migration assay. Stained with 0.1% crystal violet (400×); (**E**) The cell migration was determined with wound healing test. Data are shown as mean ± SD. * *p* < 0.05, ** *p* < 0.01, *** *p* < 0.001, vs. control groups.

To confirm the effect of BMP9 on bladder cancer cells, the MTT (3-(4,5-dimethyl-2-thiazolyl)-2,5-diphenyl-2-*H*-tetrazolium bromide) assay, the colony forming test, and the EDU (5-ethynyl-2′-deoxyuridine) test were used to determine the cell proliferation changes. As shown in Figure 2A–C, the BIU-87 cell proliferation was significantly increased after being up-regulated by BMP9. In contrast, inhibited cell proliferation was observed in T24 cells by silencing BMP9. Afterward, the cell migration changes were measured using the Transwell migration assay and the wound-healing test. Results showed that BMP9 overexpression enhanced the migration in BIU-87 cells while silencing BMP9 reduced the migration in T24 cells (see Figure 2D,E).

### 2.3. BMP9 Up-Regulate the Expression of lncRNA UCA1 in Bladder Cancer Cells 

**Figure 3:**
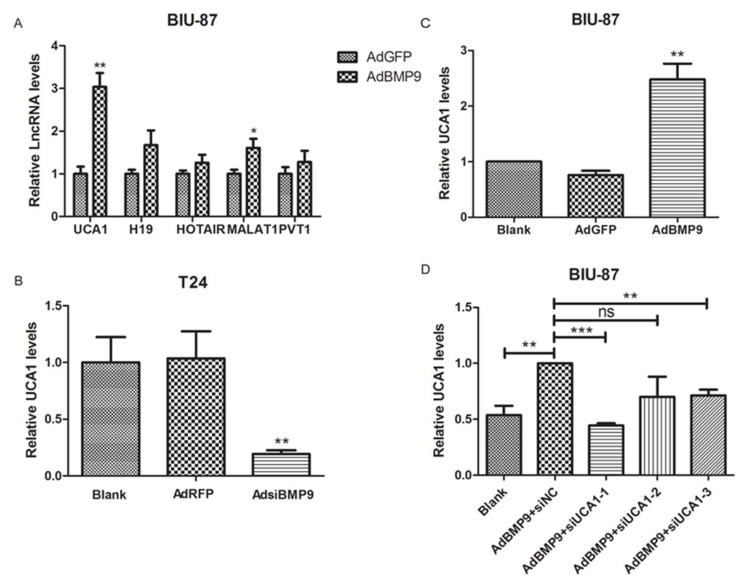
BMP9 up-regulated the expression of lncRNA UCA1 in bladder cancer cells. (**A**) Five common lncRNA were screened in BIU-87 cells after transfected with AdBMP9 by RT-PCR; (**B**) The expression of lncRNA UCA1 were verified in BIU-87 cells after transfected with AdBMP9 by RT-PCR; (**C**) The expression of lncRNA UCA1 were tested in T24 cells after being transfected with AdsiBMP9 by RT-PCR; (**D**) The inhibitory effect of siUCA1 were analyzed by RT-PCR in BIU-87 cells after being co-transfected with AdBMP9 and siUCA1. Data are shown as mean ± SD. ns *p* > 0.05, * *p* < 0.05, ** *p* < 0.01, *** *p* < 0.001, vs. control groups.

5 lncRNAs, which were reported to be closely related to bladder cancer, were screened and verified by RT-PCR. As shown in Figure 3A, the *UCA1* was the most clearly up-regulated lncRNA after transfection with AdBMP9 in BIU-87 cells. In the subsequent verification test, a positive correlation between BMP9 and *UCA1* was also found in BIU-87 and T24 cells (see Figure 3B,C). To investigate the role of *UCA1* in the effect of BMP9 on bladder cancer cells, the small interference RNA targeting *UCA1* (siUCA1) was synthesized and the interference efficiency of these three siUCA1s was verified by RT-PCR. As shown in Figure 3D, after being co-transfected with AdBMP9 and siUCA1, the siUCA1-1 has a significant inhibitory effect on *UCA1* and was used in the following tests.

### 2.4. BMP9 Promotes the Proliferation and Migration of Bladder Cancer BIU-87 Cells through UCA1 

**Figure 4:**
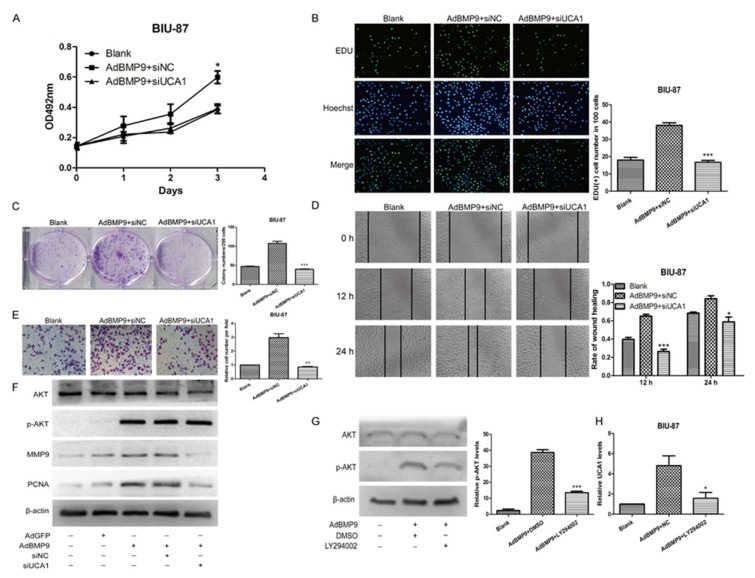
BMP9 promotes the proliferation and migration through up-regulating *UCA1*. (**A**) The cell proliferation was detected by MTT assay; (**B**) The cell proliferation was detected by EDU test. Green for proliferation cells nucleus. Blue for all living cells nucleus (400×); (**C**) The cell proliferation was detected by colony forming test. Stained with 0.1% crystal violet; (**D**) The cell migration was determined with wound healing test; (**E**) The cell migration was determined with the Transwell migration assay. Stained with 0.1% crystal violet (400×); (**F**) The expression levels of AKT, p-AKT, MMP9, and PCNA in bladder cancer cells were detected by western blot; (**G**) Verification of inhibition effect of PI3K-AKT inhibitor LY294002; (**H**) RT-PCR showed the effect of LY294002 on *UCA1* in BMP9 overexpressed BIU-87 cells. Data are shown as mean ± SD. * *p* < 0.05, ** *p* < 0.01, *** *p* < 0.001, vs. control groups.

To investigate whether *UCA1* plays a role in the effect of BMP9 on bladder cancer cells, the MTT assay, the colony forming test, and the EDU test were used to test the proliferation of BIU-87 cells after being co-transfected with AdBMP9 and siUCA1. The wound-healing test and the Transwell migration assay were used to determine the migration as well. We found that the promoting effect of BMP9 on bladder cancer BIU-87 cells was significantly rescued by siUCA1 (see Figure 4A–E). Afterward, the protein levels of proliferating cell nuclear antigen (PCNA) and matrix metalloprotein-9 (MMP-9) in BIU-87 cells were detected by western blot. As shown in Figure 4F, PCNA and MMP-9 were both increased after being transfected with AdBMP9 and were rescued by siUCA1, which is consistent with the previous phenomenon. In our antecedent experiments, we found that BMP9 could play roles through the PI3K-AKT signaling pathway [26]. Therefore, we detected the expression of AKT and p-AKT in BIU-87 cells. The level of phosphorylated AKT (p-AKT) protein was significantly increased by BMP9 while the total AKT was not significantly changed (see Figure 4F). This indicated that p-AKT may also be a downstream factor when it comes to the effect of BMP9 on bladder cancer cells. To verify this supposition, we treated the BIU-87 cells with the AKT inhibitor LY294002 after being transfected with AdBMP9 (see Figure 4G) and then detected the expression of *UCA1* by using RT-PCR. As shown in Figure 4H, the expression of *UCA1* was rescued by LY294002 in BMP9 overexpressed in BIU-87 cells given that interference of *UCA1* could not affect the phosphorylation of AKT (see Figure 4F), which suggests that the BMP9 may increase the expression of *UCA1* by promoting the phosphorylation of AKT. These data demonstrate that the BMP9-p-AKT-*UCA1* axis promotes the proliferation and migration of bladder cancer cells.

### 2.5. BMP9 Promotes the Proliferation and Migration of Bladder Cancer Cells in a UCA1 Dependent Way in Vivo

**Figure 5:**
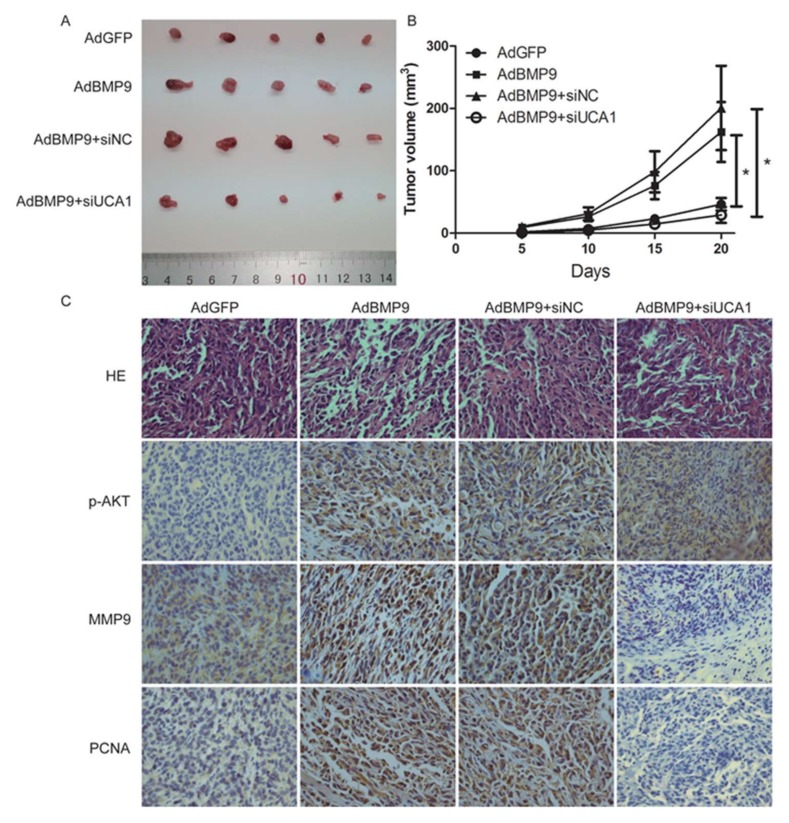
BMP9 promotes the proliferation and migration of bladder cancer cells through UCA1 in vivo. (**A**) The tumors were excised after three weeks; (**B**) Tumor growth curve of different groups. Data are shown as mean ± SD (* *p* < 0.05); (**C**) HE and immunohistochemistry of tumors in different groups (×400).

To further investigate the effect of BMP9 on the proliferation and migration of bladder cancer cells in vivo, we established a xenograft model in nude mice. 5 × 10^6^ BIU-87 cells were inoculated subcutaneously in five-week-old male, nude mice after treating with AdBMP9 and siUCA1. Tumor volume was measured once every five days. As shown in Figure 5A,B, the tumor volume was larger in the AdBMP9 group than in the AdGFP group (*p* < 0.05) and this trend was significantly weakened after transfection of siUCA1 (*p* < 0.05). Consistent with previous observations, immunohistochemistry showed that the protein levels of p-AKT, MMP9, and PCNA were increased after transfection of AdBMP9. This promotion was rescued after reducing the expression of *UCA1*, except for p-AKT (see Figure 5C). These data demonstrate that BMP9 promotes the proliferation and migration of bladder cancer cells through *UCA1*.

## 3. Discussion

As the most common malignant tumor of the urinary system, the treatment of bladder cancer is mainly done by surgical resection of primary focus and postoperative chemotherapy. However, due to the occult of bladder cancer, the newly diagnosed bladder cancer is often accompanied by muscle infiltration or even distant metastasis [27,28,29]. High metastasis and the recurrence rate were the main causes of high mortality of bladder cancer. Statistics show that 50% of patients with muscle invasive bladder cancer have metastasis and death within three years after diagnosis [30,31]. Therefore, it is particularly important for exploring the mechanism of the growth and metastasis of bladder cancer.

Transforming the growth factor-β (TGF-β) is known to promote tumor proliferation and migration. Bone morphogenetic proteins (BMPs) and members of the TGF-β superfamily have been confirmed to be involved in a variety of biological activities. BMP9, a member of the BMP family, is reported to be closely related to the development of the tumor. For instance, BMP9 could promote the proliferation and migration of liver cancer cells but also could inhibit the growth of breast cancer and osteosarcoma cells [32]. However, the expression of BMP9 in bladder cancer cells and its role in bladder cancer have not yet been reported. We found that the expression of BMP9 in the bladder cancer cells was significantly higher than in normal bladder cells. Considering that BMP2 and BMP7 could promote the proliferation and migration of bladder cancer, we try to confirm whether BMP9 plays a role in the development of bladder cancer.

In this study, we first measured the basic expression levels of BMP9 in two bladder cancer cells as well as T24 and BIU-87 cells and found that BMP9 was highly expressed in T24 cells. Afterward, we enhanced the expression of BMP9 in BIU-87 cells and reduced it in T24 cells by using the recombinant adenovirus. We used the MTT assay, the colony forming test, and the EDU test to analyze cells proliferation. The wound-healing test and Transwell migration test were used to detect the cells migration. We found that up regulation of the expression of BMP9 can promote the proliferation and migration of bladder cancer cells while down regulation of its expression can inhibit the proliferation and migration of bladder cancer cells.

In order to determine how BMP9 is implicated in bladder cancer cells, we focused on lncRNA since it has been proven that the dysregulation of lncRNA may affect the occurrence and development of bladder cancer [33]. We detected five common types of lncRNAs reported to be closely related to bladder in the BIU-87 cells after being transfected with AdBMP9 and found that lncRNA *UCA1* was significantly increased in the AdBMP9 group. *UCA1* is a recently identified long non-coding RNA and characterized as a sensitive and specific marker for human bladder cancer. It is highly expressed in bladder cancer cells while the mechanism of high expression is not clear. In our study, we constructed a cell model that co-transfected AdBMP9 and siUCA1 in bladder cancer BIU-87 cells and then observed the proliferation and migration changes. We found that the promoting effect was rescued after interfering with *UCA1*, which indicated that the effect of BMP9 on bladder cancer cells was achieved through *UCA1*. The result of Western blot for markers of proliferation and migration also proved this result.

Then we explored the mechanism that BMP9 promotes the expression levels of *UCA1*. In our previous study, we found that BMP9 plays roles in classic Smad or non-Smad signaling cascades including the PI3K/AKT and p38MAPK pathways. Consider that *UCA1* is associated with the AKT signal pathway [34,35] and that there is a correlation between *UCA1* and AKT in the gene cards database. We decided to verify whether there is a relationship between BMP9, AKT, and *UCA1*. After being transfected with AdBMP9, the level of AKT was maintained while the phosphorylation at the S473 site of AKT was elevated, which means that AKT is activated. Furthermore, the expression of *UCA1* was decreased after being treated with the AKT inhibitor in BMP9 over expressed BIU-87cells. These results show that BMP9 can increase the expression of *UCA1* by activating AKT.

Finally, the subcutaneous transplanted tumor test in male nude mice showed the same results as above. BMP9 remarkably increased the growth of transplanted tumor in a *UCA1* dependent way. The immunohistochemistry of p-AKT, MMP9, and PCNA of the xenograft tumor were also consistent with the previous experiments. One puzzling result of the immunohistochemistry was that PCNA were positive in both cytoplasma and nucleoplasm whereas it should only be located in the nuclear. This result may be attributed to the lower specificity of the PCNA polyclonal antibody.

Taken together, our study confirmed that BMP9 can increase the expression of *UCA1* through phosphorylated AKT, which promotes the proliferation and migration of bladder cancer cells. It is suggested that BMP9 can be used as a novel diagnostic marker and a potential therapeutic target for bladder cancer.

## 4. Materials and Methods

### 4.1. Cell Culture

Human bladder cancer BIU-87 and T24 cells were obtained from the China Center for Type Culture Collection (CCTCC). Both of the cells were maintained in Dulbecco’s modified Eagle’s medium (DMEM; HyClone Laboratories, Logan, UT, USA) with 10% fetal bovine serum (FBS; Gibco, USA), 100 U/mL penicillin, and 100 μg/mL streptomycin. The cultures were grown at 37 °C with 5% CO_2_.

### 4.2. Recombinant ADENOVIRUSES and siRNA Transfection 

Recombinant adenoviruses BMP9 (AdBMP9), recombinant adenoviruses green fluorescent protein (AdGFP), recombinant adenoviruses small interfering BMP9 (AdsiBMP9), and recombinant adenoviruses red fluorescent protein (AdRFP) were obtained from University of Chicago Medical Center (University of Chicago, Chicago, IL, USA).

The siUCA1 and negative control (siNC) were purchased from Shanghai GenePharma Co., Ltd. (Shanghai, China). The sequences are shown in Table 1. The siRNA transfection reagent Entranster^TM^-R4000 was purchased from Engreen Biosystem Co., Ltd. (Beijing, China).

### 4.3. Western Blot Analysis 

The protein was extracted from cells after three days culture and lysed in RIPA (Radio Immunoprecipitation Assay) buffer (Beyotime Institute of Biotechnology, Shanghai, China). After being separated by 10% SDS-polyacrylamide gel electrophoresis and membrane transfer, the PVDF (polyvinylidene fluoride) membranes were blocked with 5% bovine serum albumin at 37 °C for 2 h. Next, the membranes were incubated with the primary antibodies against BMP9 (1:500; Abcam, Cambridge, MA, USA); AKT (1:1000; Wanleibio Co., Ltd., Beijing, China); p-AKT (Ser473) (1:1000; Cell Signaling Technology, Danvers, MA, USA); PCNA (1:1000; Wanleibio Co., Ltd., Beijing, China); MMP9 (1:500; Cell Signaling Technology, Danvers, MA, USA), and β-actin (1:1000; Santa Cruz Biotechnology, CA, USA). This was followed by treatment with HRP-conjugated secondary antibody (1:5000; Beijing Zhongshan Golden Bridge Biotechnology) for 1 h at 37 °C. The protein bands were visualized with the Supersignal West Pico Chemiluminescent substrate kit (Millipore, Billerica, MA, USA).

### 4.4. Real-Time PCR Analysis 

The RNA was extracted by TRIzol reagent (Invitrogen, Carlsbad, CA, USA) according to the manufacturer’s protocol. 1.5 μg of RNA was reverse transcribed to cDNA with Reverse transcription reagent kit (TaKaRa Co., Dalian, China). The lncRNA *UCA1* expression was normalized with *β-actin*. Primers are shown in Table 1.

### 4.5. MTT Assay

The cell viability was tested by MTT [3-(4,5-dimethylthiazol-2-yl)-2,5-diphenyltetrazolium bromide] colorimetric assay. The 3000 cells per well were seeded in 96-well plate and cultured for 0, 24, 48, and 72 h after transfection. Additionally, 20 μL MTT reagent (5 mg/mL; Sigma-Aldrich, St. Louis, MO, USA) was transferred into each well and incubated for 4 h. Afterward, 150 μL dimethylsulfoxide was added into each well after the cell culture medium was abandoned. Finally, the absorbance was measured at 492 nm with the use of a microplate reader.

### 4.6. Colony Forming Test

In addition, 1000 cells per well were seeded into 6-well plate with 1% FBS medium after transfection for 8 days. Afterward, the cells were fixed with 4% paraformadehyde and stained with 0.1% crystal violet.

### 4.7. EDU Test

In this test, 3000 cells per well were seeded into a 96-well plate, the EDU reagent and Hoechst reagent (RiboBio Co., Ltd.; Guangzhou, China) were used to stain the cells one day after transfection. The EDU(+)/Hoechst(+) presents the proportion of the proliferating cells.

### 4.8. Wound-Healing Test

The cells were seeded into a 6-well plate and it grew by 90% after transfection. A wound was made with a 10 μL pipette tip and culture for 24 h. The specific wound area was photographed at the time points of 0, 12, and 24 h, respectively. The wound-healing rate was calculated as follows: (0 h width − 12 h or 24 h width)/0 h width × 100%.

### 4.9. Transwell Migration Assay

After transfection for 1 day, 20,000 cells per well were collected to seed into the transwell upper chamber with 200 μL 0% culture medium. In addition, 600 μL 10% FBS culture medium were transferred into the transwell lower chamber. The cells were cultured for 24 h and fixed with 4% paraformaldehyde and stained with 0.1% crystal violet.

### 4.10. Xenograft Model

After transfection for 1 day, 5 × 10^6^ cells per tumor were inoculated subcutaneously into male nude mice (5–6 weeks) and grown for 3 weeks. The nude mice were sacrificed by cervical vertebra dislocation. The tumor was exposed to immunohistochemistry.

### 4.11. Immunohistochemistry

The tumor tissues were embedded with paraffin and sectioned to 5 μm. The sections were incubated with primary antibody at 4 °C overnight after antigen retrieval. Then, the sections were performed with an immunohistochemistry SP-9000 kit (Beijing Zhongshan Golden Bridge Biotechnology, Co., Ltd.; Beijing, China).

### 4.12. Statistical Analysis

All the experiments were repeated three times and analyzed with GraphPad Prism (Prism 5.0, GraphPad Software, San Diego, CA, USA). The student’s *t*-test was used to evaluate the differences between two groups. The *p* < 0.05 was considered statistically significant. * *p* < 0.05, ** *p* < 0.01, *** *p* < 0.001.

## Figures and Tables

**Table 1:** Primers and siRNA sequences.

Targets		Sequence
*β-actin*	Forward	5′-CACCACACCTTCTACAATGAGC-3′
	Reverse	5′-GTGATCTCCTTCTGCATCCTGT-3′
*UCA1*	Forward	5′-CTCTCCATTGGGTTCACCATTC-3′
	Reverse	5′-GCGGCAGGTCTTAAGAGATGAG-3′
*H19*	Forward	5′-ATCGGTGCCTCAGCGTTCGG-3′
	Reverse	5′-CTGTCCTCGCCGTCACACCG-3′
*HOTAIR*	Forward	5′-CCAGTTCTCAGGCGAGAGCC-3′
	Reverse	5′-TTTATATTCAGGACATGTAA-3′
*MALAT1*	Forward	5′-ATGCGAGTTGTTCTCCGTCT-3′
	Reverse	5′-TATCTGCGGTTTCCTCAAGC-3′
*PVT1*	Forward	5′-GCCCCTTCTATGGGAATCACTA-3′
	Reverse	5′-GGGGCAGAGATGAAATCGTAAT-3′
siUCA1-1	Forward	5′-GGGCUUGGGACAUUUCACUTT-3′
	Reverse	5′-AGUGAAAUGUCCCAAGCCCTT-3′
siUCA1-2	Forward	5′-GAGCCGAUCAGACAAACAATT-3′
	Reverse	5′-UUGUUUGUCUGAUCGGCUCTT-3′
siUCA1-3	Forward	5′-GGGAAUACUAUUCGUAUGATT-3′
	Reverse	5′-UCAUACGAAUAGUAUUCCCTT-3′
siNC	Forward	5′-UUCUCCGAACGUGUCACGUTT-3′
	Reverse	5′-ACGUGACACGUUCGGAGAATT-3′
